# The relationship between serum levels of fibroblast growth factor 21 and diabetic retinopathy

**DOI:** 10.17179/excli2017-672

**Published:** 2017-11-22

**Authors:** Zohre Mousavi, Shokoufeh Bonakdaran, Amirhossein Sahebkar, Gholamhossein Yaghoubi, Mohammad Ali Yaghoubi, Najmeh Davoudian, Masoud Mohebbi

**Affiliations:** 1 Endocrine Research Center, Mashhad University of Medical Sciences, Mashhad, Iran; 2 Biotechnology Research Center, Pharmaceutical Technology Institute, Mashhad University of Medical Sciences, Mashhad, Iran; 3 School of Pharmacy, Mashhad University of Medical Sciences, Mashhad, Iran; 4 Birjand University of Medical Science, Birjand, Iran

**Keywords:** fibroblast growth factor 21, type 2 diabetes mellitus, diabetic retinopathy

## Abstract

Fibroblast growth factor 21 (FGF21) is a major metabolic regulator that has been shown to be elevated in a number of metabolic disturbances including type 2 diabetes mellitus (T2DM) and the metabolic syndrome, but few studies about the relationship between serum FGF21 and the complications of diabetes have been done. Since the association between FGF21 and diabetic retinopathy is not clear, this study was conducted to investigate this relationship. In this cross-sectional study, 61 subjects (14 healthy controls, 22 diabetic patients without retinopathy, and 25 patients with diabetic retinopathy) were evaluated. All patients in the study were examined for the presence of diabetic retinopathy. Various clinical and biochemical parameters including FGF21 were evaluated and analyzed and compared between the study groups. Serum levels of FGF21 showed a significant difference between the three groups (*P=*0.003) but the difference between diabetic patients with and without retinopathy was not significant (*P=*0.122). Regression model was used to evaluate the role of FGF21 in predicting diabetic retinopathy. In the multivariate logistic regression model after adjustment of systolic blood pressure and fasting blood glucose, the level of FGF21 was not associated with diabetic retinopathy. In the multivariate model, only fasting blood glucose was associated with diabetic retinopathy (*P=*0.009). According to the results of this study, serum levels of FGF21 in diabetic patients was higher than the control group but these raised levels could not predict the presence of diabetic retinopathy.

## Introduction

Despite great improvement in the control of diabetes, the incidence of blindness among diabetic patients is increasing (Gunduz and Bakri, 2007[7]; Abbate et al., 2011[1]). Diabetic retinopathy (DR) is a leading cause of visual impairment in patients at productive age (Priscakova et al., 2016[13]). Loss of vision in DR can be secondary to macular edema (ME), bleeding from new vessels, retinal detachment or neovascular glaucoma. Diabetic retinopathy can be classified as non-proliferative and proliferative. Features of non-proliferative DR (NPDR) include microaneurysms of retinal vessels, blot hemorrhages and bleeding spots on the retina. In these patients, capillary wall permeability increases and may cause bleeding in the retina of different levels. Proliferative DR (PDR) causes more severe ocular complications of diabetes. In this type of retinopathy neovascularization at the vitreoretinal interface occurs due to ischemia (Frank, 2004[6]).

Many growth factors are involved in the development of DR, including vascular endothelial growth factor (VEGF), platelet-derived growth factor (PDGF), insulin-like growth factor (IGF) and fibroblast growth factors (FGF) (Zakareia et al., 2010[17]; Praidou et al., 2011[12]). The role of these factors in the pathophysiology of DR is complex. Fibroblast growth factor family contains 22 members with a wide range of biological functions, including involvement in cell growth, development, production and proliferation of blood cells and healing of the wounds (Belov and Mohammadi, 2013[2]).

FGF21 is a member of the FGF group that is constitutively secreted from the liver and increases glucose uptake in adipocytes by enhancing the expression of glucose transporter 1 (Glut1) (Canto and Auwerx, 2012[3]). Recent studies have demonstrated the role of FGF21 in the regulation of glucose and lipid metabolism as well as its possible impact on the treatment of diabetes and obesity (Smith et al., 2013[14]). It has been shown that serum FGF21 levels are higher in obese, type 2 diabetic patients and in people with metabolic syndrome (Lin et al., 2012[10]; Xiao et al., 2012[16]). To our knowledge, only few studies have assessed the relationship between FGF21 and DR, including a study in China that showed an association between higher serum levels of FGF21 with the severity of DR (Lin et al., 2014[9]). Since the association between serum FGF21 levels and retinopathy is not clear, this study was conducted to investigate this association.

## Methods

In this cross-sectional study, patients with type 2 diabetes referring to the Diabetes Clinic of the Imam Reza hospital (Mashhad, Iran) between 2016 and 2017 were selected based on the American Diabetes Association diagnostic criteria for diabetes. Inclusion criteria in this study was diabetic patients with the age of more than 25 years old and exclusion criteria were history of diabetic nephropathy or GFR < 60 mL/min, having chronic liver disease, exercise or heavy exertion three days before sampling, active infection or inflammation and consumption of drugs that have effects on immune system like corticosteroids. After completing the questionnaire, patients were evaluated in terms of developing diabetic retinopathy by an ophthalmologist (after pupil dilation, under Fundoscopy by +90 diopter lens and indirect fundoscopy). 

At the first step, the sight of two eyes (with refractive error correction) was measured separately. Indirect ophthalmoscopy was performed by +20 and +30 lenses. At last 22 patients without retinopathy and 25 patients with diabetic retinopathy who met the pre-defined inclusion criteria were enrolled to the study.

Patients without DR were those who did not have any manifestation of diabetic retinopathy like microaneurysm, cotton wool spots, retinal hemorrhage or macular edema. Patients with type 2 DR were those with type II diabetes who had manifestations of diabetic retinopathy (such as micro-aneurysms, cotton wool spots, retinal hemorrhage or macular edema), these patients were categorized into two groups: patients with non-proliferative diabetic retinopathy (NPDR) who had no evidence of neovascularization originating from the disc, retina or iris, and patients with proliferative DR (PDR) in whom there was presence of neovascularization arising from the optic disc, retina, iris and the consequences of this neovascularization, such as vitreous hemorrhage.

A total of 14 healthy control people who came to the clinic with the patients and had fasting blood glucose < 100 mg/dl were enrolled into the study using convenience sampling as the control group. After explaining the purpose of the study and obtaining informed consent, participants were enrolled and a questionnaire about demographic characteristics (age, sex, education, occupation) and family history of hypertension and diabetes was completed. The weight of all participants was measured using digital scale (Seca, Germany) with minimal cover and the empty bladder. Standing height of the patients was measured by the meter without shoes. Body mass index (BMI) was calculated by dividing weight (kg) by the square of height (m). Blood pressure was measured according to the WHO guideline using a mercury sphygmomanometer with a cuff of appropriate size twice at an interval of 10-15 minutes and mean blood pressure was recorded. Then participants were introduced to the laboratory for testing fasting blood glucose, hemoglobin A1C, blood lipid profile (triglycerides, cholesterol, LDL, and HDL), urea, creatinine, urinalysis for proteinuria and FGF21.

All of the patients were in a fasting status for 12 h at the time of blood sampling and 5 mL of venous blood from a brachial vein was collected. Serum level of FGF21 was assessed using an ELISA kit (Biovendor LLC, with 2 % intra-assay and 3.3 % intra-assay variations). Blood glucose, hemoglobin A1C and lipid profiles were measured using routine commercial kits (Pars Azmoon Co., Iran).

### Statistical analysis

Data were analyzed using SPSS software version 16.0 (SPSS Inc., Chicago, IL, USA). Continuous variables were expressed as mean ± standard deviation and categorical variables as number and percentage. Independent samples *t*-test and Mann-Whitney U tests were used for comparison of quantitative variables between two groups. Kruskal-Wallis and ANOVA tests were used to compare means among three groups and the chi-square test was used for comparison of categorical variables. Spearman's correlation test was used to assess the association between quantitative variables and FGF21. A two-tailed *P* value of less than 0.05 was considered as statistically significant. Univariate logistic regression and multivariate logistic regression analyses were performed to test the effect of FGF21 (ORs) adjusted for FBS and systolic blood pressure in predicting DR. The role of FGF21in predicting DR was also analyzed using receiver operating characteristic (ROC) curve and the optimal cut-off was determined using the Youden index.

## Result

Clinical and laboratory findings of the participants are shown in Table 1(Tab. 1). There was no significant difference in gender, age and BMI between controls and diabetic patients with and without retinopathy. The median duration of diabetes was significantly higher in diabetic patients with retinopathy compared with patients without DR. There was no significant difference in the prevalence of the positive family history of diabetes, and consumption of anti-hypertensive drugs (angiotensin-converting enzyme inhibitors or angiotensin receptor blockers) between type 2 diabetic patients with and without DR. Systolic and diastolic blood pressure, urea, creatinine, triglycerides, total cholesterol, LDL-C, HDL-C, ALT and AST showed no significant difference between controls and diabetic patients with and without retinopathy. However, there was a significant difference in FBS and HbA1c levels among the three groups (P<0.001) and all *post-hoc* comparisons revealed significant differences (P<0.05). The result of the Kruskal-Wallis test showed a significant difference between serum FGF21 levels of the controls and diabetic patients with and without retinopathy (*P=*0.003). *Post-hoc* analysis using Jonckheere-Terpstra test showed no significant difference between FGF21 levels of the two groups of diabetic patients with and without retinopathy (*P=*0.122).

Serum levels of FGF21 were similar in diabetic patients without retinopathy and control group (*P=*0.112). Also, there was no significant difference between the levels of FGF21 in patients with different degree of DR (*P=*0.287). Serum levels of FGF21 in control group and diabetic patients and in patients with different level of diabetic retinopathy are shown in Table 2(Tab. 2).

Body mass index showed a significant correlation with FGF21 (r=0.285 and *P=*0.026). Results also showed a positive correlation between serum levels of triglycerides and FGF21 (r=0.385, *P=*0.002). Correlations between systolic blood pressure, HDL, and FBS with FGF21 were marginally significant (Table 3(Tab. 3)). For multivariate regression model, variables which had significant or marginally significant correlations with FGF21 were entered into the model.

The model was used to evaluate the capability of FGF21 to predict diabetic retinopathy. According to the results of univariate logistic regression model HbA1c, FBS and FGF21 had significant associations with the risk of diabetic retinopathy. Each unit increase in the level of HbA1c was associated with 2 % increase in the risk of DR. In the multivariate model, HbA1c was removed due to its high correlation with FBS (r=0.848 and P<0.001).

In the multivariate logistic regression model, after adjustment for potential confounding variables, i.e. systolic blood pressure and FBS, the level of FGF21 was not associated with DR. In the multivariate model, only FBS was associated with DR (*P=*0.009) and each one unit increase in the level of FBS was associated with 9.1 % increase in the risk of DR **(**Table 4(Tab. 4)).

**Figure 1:**
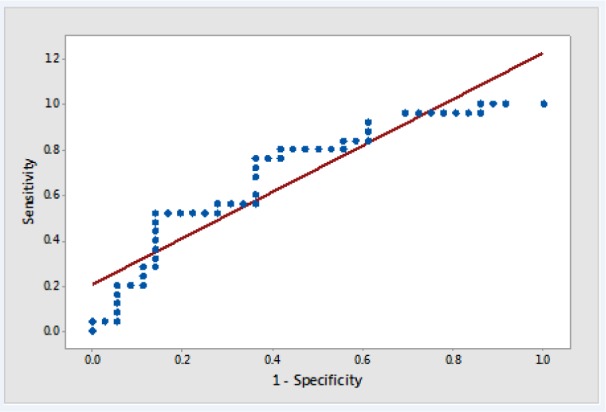
Roc curve for serum FGF21 in predicting the occurrence of diabetic retinopathy

To evaluate the accuracy of FGF21 in the prediction of DR, ROC curve analysis was used (Figure 1(Fig. 1)). Area under the curve in this model was 71.1 % and optimal cut-off point was calculated at 196 (pg/mL), with a sensitivity of 80 % and specificity of 47.2 %.

See also the Supplementary data file.

## Discussion

Studies have shown that FGF21 is a protein with hepatic origin and broad biological roles in humans and animals (Woo et al., 2013[15]). FGF21 is released as a signal for starvation and leads to gluconeogenesis, lipoprotein oxidation and lipolysis in fat tissues. While FGF21 is expressed through PPAR cascade, its biological actions are mediated through binding to a complex formed by its receptors and an essential coreceptor, β-Klotho (Cheung and Deng, 2014[4]). Defects in the expression or activation of FGF21 lead to decreased sensitivity to insulin, hepatic lipid oxidation, and impaired clearance of triglycerides in some cases such as PPAR signaling disorders (Liu et al., 2015[11]). On the other hand, increased concentrations of FGF21 can be observed in several pathological states such as insulin resistance, obesity, hypertension, diabetes, and metabolic syndrome, which may indicate that increased FGF21 is a response to unfavorable metabolic conditions (Woo et al., 2013[15]; Cheung and Deng, 2014[4]).

An increase in serum FGF21 concentrations has been observed in patients with micro-vascular and macro-vascular complications (Lee et al., 2015[8]). In our study, comparison of serum FGF21 levels among healthy controls, diabetic patients without retinopathy and patients with DR showed a significant difference. However, the FGF21 difference between the two groups of diabetic patients with and without retinopathy was not significant. In the multivariate model analysis, it was found that after adjustment for systolic blood pressure and FBS, the level of FGF21 was not associated with DR while each unit increase in FBS increased the likelihood to develop DR by 1.9 percent, and FBS was the only significant variable in the multivariate model. Moreover, based on the results of this study, there was a significant positive correlation between serum levels of FGF21 with BMI and triglycerides.

According to the study of Lin and colleagues (Lin et al., 2014[9]), mean FGF21 was significantly higher in diabetic patients than the control group. However, unlike our study in which there was no difference between patients with and without retinopathy, mean FGF21 in patients without retinopathy was significantly lower than patients with proliferative and non-proliferative retinopathy. In logistic regression analysis after adjusting for HOMA-IR, insulin, HbA1c and glucose, FGF21 remained significantly associated with diabetes.

According to the study of Esteghamati and colleagues (Esteghamati et al., 2016[5]), serum levels of FGF21 in patients with and without DR were significantly higher than the control group. However, in their study, serum levels of FGF21 was significantly lower in type 2 diabetic patients without retinopathy than the patients with DR and according to the regression model, FGF21 was predictive of increasing DR risk after adjustment for triglycerides levels and disease period.

Differences in the findings of above-mentioned studies can be due to different numbers of the patients since Lin and colleagues (2014[9]) studied 111 diabetic patients (49 with proliferative retinopathy and 34 with non-proliferative retinopathy). On the other hand, Esteghamati and colleagues studied 90 diabetic patients of whom 46 cases had DR, while in our study there were 14 participants in the control group, 22 in the non-retinopathy diabetic group, and 25 in the DR group. It should be mentioned that the sample size in our study was determined based on the mean difference of FGF21 between diabetic and non-diabetic patients and, therefore, it might have been smaller than the required size to detect a significant difference in serum FGF21 levels between patients with and without DR or between patients with various intensities of DR. Another important point to explain the discrepant findings of the study by the study of Lin and colleagues (Lin et al., 2014[9]) relates to the difference in the screening of diabetes in China, where diabetic patients are identified in the initial stages and through an appropriate screening program. 

In the studies by Lin et al. (2014[9]) and Esteghamati et al. (2016[5]), no significant difference was found in serum FGF21 levels between patients with proliferative and non-proliferative DR. However, in the study of Esteghamati et al. (2016[5]), patients with severe non-proliferative retinopathy (diabetic macular edema for example) were included in the proliferative retinopathy group due to the high risk of the disease progressing to loss of vision, while this was not the case in our study as well as in the study by Lin et al. (2014[9]).

According to the results of ROC analysis, the best cut-off point of serum FGF21 levels was estimated as 196 pg/mL which is significantly lower than that estimated by Lin et al. (2014[9]). In the study of Lin and colleagues, mean serum levels of FGF21 in the control group, diabetic patients without retinopathy, patients with non-proliferative retinopathy, and patients with proliferative retinopathy were respectively 125.9, 326.8, 631.9, and 669.4 pg/mL, respectively. Use of lower cut-off points such as 196 pg/mL obtained in the study or 135 obtained by Esteghamati et al. (2016[5]) has the advantage that patients with progressing diabetes who are at risk of retinopathy are identified with a higher sensitivity. Moreover, based on ROC model, 71.1 % accuracy was calculated for serum levels of FGF21 in the identification of patients with retinopathy.

The present study had several limitations. For example, the study was performed on a limited number of patients and although the number of diabetic and non-diabetic patients was sufficient to compare FGF21 levels, the ability of statistical test decreased in the establishment of a relationship between disease severity and FGF21 levels. Another issue was associated with the study design which was cross-sectional and did not allow an interpretation on the causal role of high concentrations of FGF21 in diabetes and DR. 

## Conclusion

Findings of the present study indicated that serum FGF21 levels were significantly higher in diabetic patients compared to the control group, but difference in serum FGF21 levels was not significant between two diabetic groups with and without retinopathy. Moreover, based on the results of this study, there was a significant positive correlation between serum levels of FGF21 with BMI and triglycerides levels. Future studies are warranted to confirm the present results in larger populations.

## Funding

This article has been extracted from the thesis of Dr. Mohammad Ali Yaghoubi (grant no. 950105) and sponsored by the Research Council at the Mashhad University of Medical Sciences (Mashhad, Iran). Experiments associated with the project were done in collaboration with the Mashhad Laboratory of Pathobiology.

## Conflict of interests

There is no conflict of interests.

## Supplementary Material

Supplementary data

## Figures and Tables

**Table 1:**
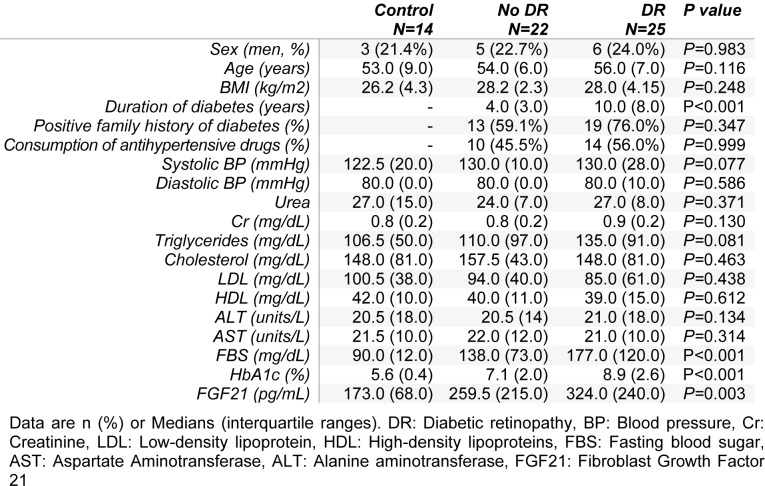
Baseline clinical and laboratory findings of the participants in the study

**Table 2:**
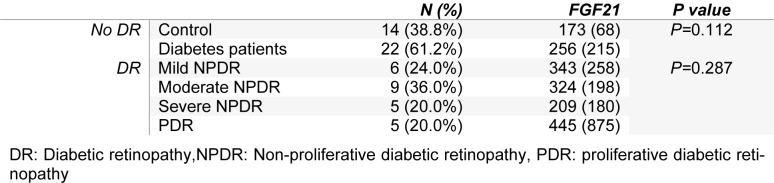
Comparison of the level of FGF21 between controls and diabetic patients and between the patients with different level of diabetic retinopathy

**Table 3:**
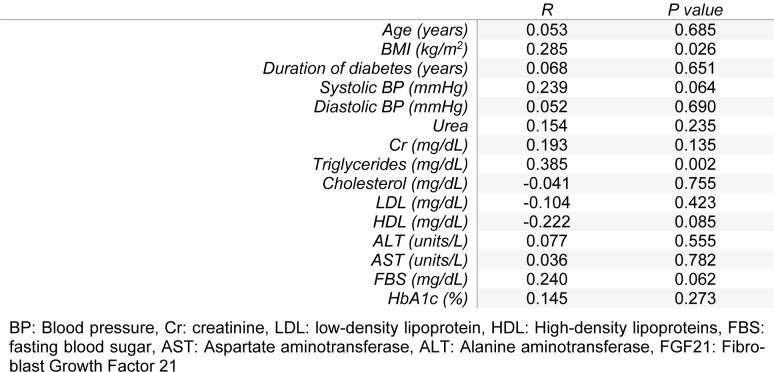
Analysis of the correlation between quantitative variables and FGF21

**Table 4:**
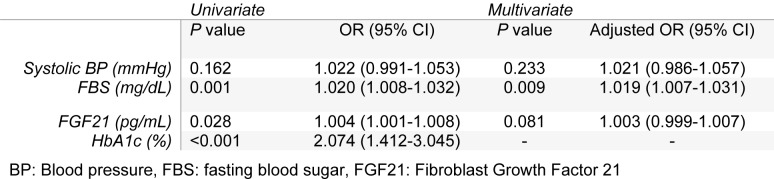
Univariate and multivariate logistic regression models for identifying determinants of type 2 diabetic retinopathy
